# Adipose-derived mesenchymal stem cells markedly attenuate brain infarct size and improve neurological function in rats

**DOI:** 10.1186/1479-5876-8-63

**Published:** 2010-06-28

**Authors:** Steve Leu, Yu-Chun Lin, Chun-Man Yuen, Chia-Hung Yen, Ying-Hsien Kao, Cheuk-Kwan Sun, Hon-Kan Yip

**Affiliations:** 1 Division of Cardiology, Department of Internal Medicine; Chang Gung Memorial Hospital-Kaohsiung Medical Center, Chang Gung University College of Medicine, Kaohsiung, Taiwan; 2 Division of Neurosurgery, Chang Gung Memorial Hospital-Kaohsiung Medical Center, Chang Gung University College of Medicine, Kaohsiung, Taiwan; 3 Department of Life Science, National Pingtung University of Science and Technology, Pingtung, Taiwan; 4 Department of Medical Research, E-DA Hospital, I-Shou University, Kaohsiung, Taiwan; 5 Division of General Surgery, Department of Surgery, Chang Gung Memorial Hospital-Kaohsiung Medical Center, Chang Gung University College of Medicine, Kaohsiung, Taiwan; 6 Center for Translational Research in Biomedical Sciences, Chang Gung Memorial Hospital-Kaohsiung Medical Center, Chang Gung University College of Medicine, Kaohsiung, Taiwan

## Abstract

**Background:**

The therapeutic effect of adipose-derived mesenchymal stem cells (ADMSCs) on brain infarction area (BIA) and neurological status in a rat model of acute ischemic stroke (IS) was investigated.

**Methods:**

Adult male Sprague-Dawley (SD) rats (n = 30) were divided into IS plus intra-venous 1 mL saline (at 0, 12 and 24 h after IS induction) (control group) and IS plus intra-venous ADMSCs (2.0 × 10^6^) (treated interval as controls) (treatment group) after occlusion of distal left internal carotid artery. The rats were sacrificed and brain tissues were harvested on day 21 after the procedure.

**Results:**

The results showed that BIA was larger in control group than in treatment group (p < 0.001). The sensorimotor functional test (Corner test) identified a higher frequency of turning movement to left in control group than in treatment group (p < 0.05). mRNA expressions of Bax, caspase 3, interleukin (IL)-18, toll-like receptor-4 and plasminogen activator inhibitor-1 were higher, whereas Bcl-2 and IL-8/Gro were lower in control group than in treatment group (all p < 0.05). Western blot demonstrated a lower CXCR4 and stromal-cell derived factor-1 (SDF-1) in control group than in treatment group (all p < 0.01). Immunohistofluorescent staining showed lower expressions of CXCR4, SDF-1, von Willebran factor and doublecortin, whereas the number of apoptotic nuclei on TUNEL assay was higher in control group than in treatment group (all p < 0.001). Immunohistochemical staining showed that cellular proliferation and number of small vessels were lower but glial fibrillary acid protein was higher in control group than in treatment group (all p < 0.01).

**Conclusions:**

ADMSC therapy significantly limited BIA and improved sensorimotor dysfunction after acute IS.

## Background

**S**troke, a growing epidemic, is the third most frequent cause of mortality in industrialized countries [1-3]. Despite state-of-the-art therapy, clinical outcome after stroke remains poor, with many patients left permanently disabled [4]. Recently, thrombolytic therapy, a more aggressive treatment strategy, has been reported to be effective for some acute ischemic stroke (IS) patients [5,6]. However, its liberal use is hampered by a lot of limitations, including the need for early implementation within the first hours after acute IS onset [5,7-9]. Of importance is that thrombolytic therapy has been found to have a relatively high incidence of serious hemorrhagic complications [9,10]. Accordingly, finding a safe and effective therapeutic alternative for patients following acute IS, especially for those unsuitable for thrombolytic therapy, is mandatory for physicians.

Cytotherapy has recently emerged as an attractive and promising new therapeutic option for the treatment of various ischemia-related disorders, i.e. cardiovascular disease and stroke, in experimental studies [3,11-13]. Recent clinical trials have also proven its feasibility and safety [3,11-14]. However, before envisaging cell-based therapy for improving ischemia-related neurologic dysfunction, some unresolved problems still need to be clarified: 1) the ideal cell source for transplantation, 2) the most appropriate route of cell administration, and, 3) the best approach to achieve an appropriate and functional integration of transplanted cells into the host tissue [3].

Interestingly, while stem cell therapy, including bone marrow-derived mesenchymal stem cells [15-17], embryonic stem cells [14] and endothelial progenitor cells [18], have been extensively investigated in the treatment of stroke in experimental studies and, to a lesser extent, in humankind, the use of adipose-derived mesenchymal stem cells (ADMSCs) for the treatment of stroke has seldom been discussed [19]. Compared with embryonic stems cells and bone marrow-derived mesenchymal stem cells, ADMSCs have the distinct advantages of being abundant, easy to obtain with minimal invasiveness, and readily cultured to a sufficient number for autologous transplantation without ethical issue. Previous study has also demonstrated a therapeutic superiority of ADMSCs over bone marrow-derived mesenchymal stem cells in an animal model of liver injury [20]. Therefore, we suggest that cytotherapy using autologous ADMSC would be a potential clinical approach to cardiovascular or cerebral vascular disease. Accordingly, in the present study, we tested the hypothesis that ADMSC therapy is safe and effective in limiting the size of brain infarct and improving neurological function in a rat model of acute IS. We further investigated whether intravenous administration was an appropriate route for ADMSC implantation.

## Methods

### Ethics

All animal experimental procedures were approved by the Institute of Animal Care and Use Committee at our hospital and performed in accordance with the Guide for the Care and Use of Laboratory Animals (NIH publication No. 85-23, National Academy Press, Washington, DC, USA, revised 1996).

### Isolation of Adipose-Derived Mesenchymal Stem Cells from Rat

**Figure 1:**
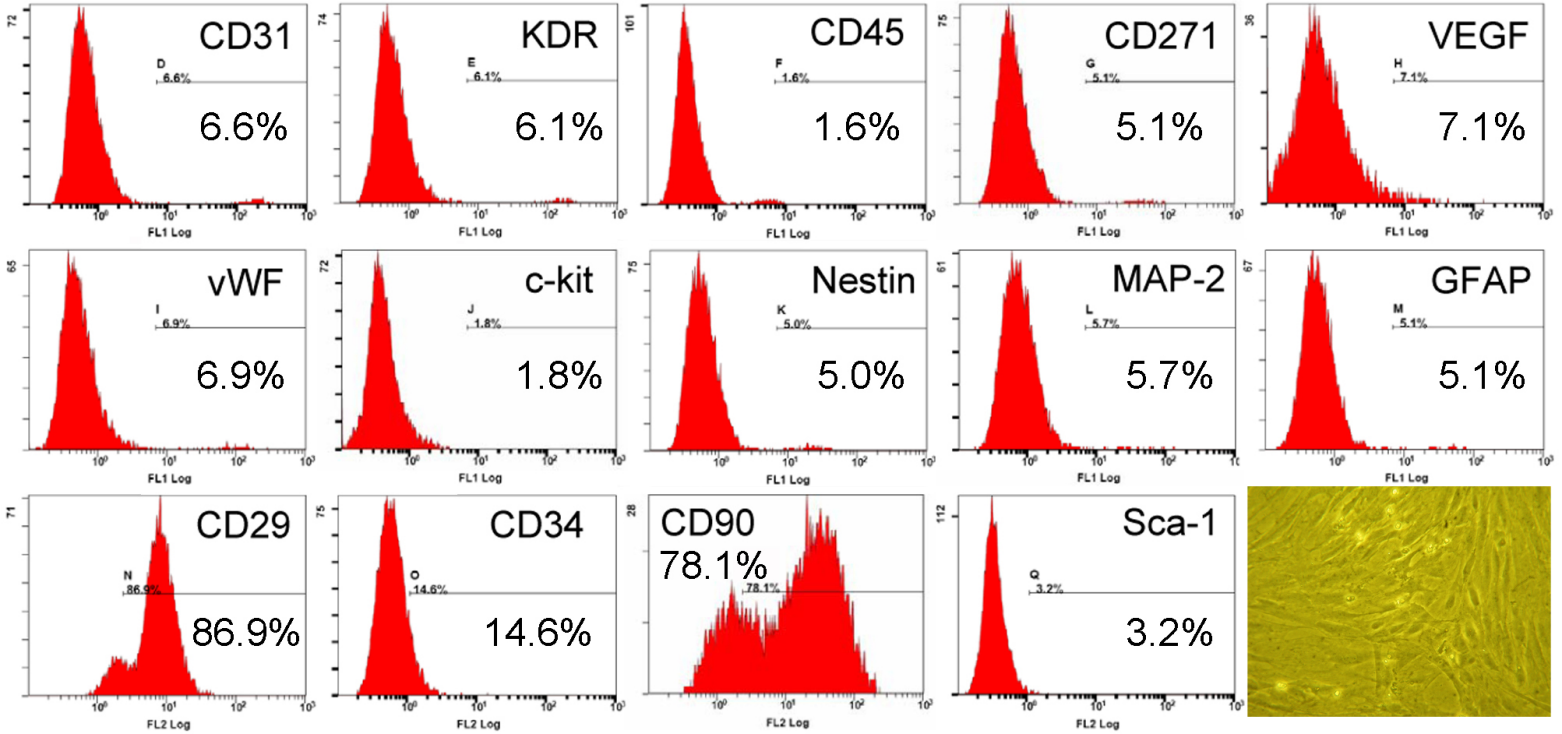
**Flow cytometric analysis of rat adipose-derived mesenchymal stem cells (ADMSCs)**. Flow cytometry results of ADMSCs (the percentage shown in figure was mean value of n = 3) on day 14 after cell culturing showed the CD29 + and CD90+ cells were the highest population of stem cells. Spindle-shaped morphological feature of the stem cells were shown in the right lower corner (200×).

The rats were anesthetized with inhalational isoflurane. Adipose tissue surrounding the epididymis was carefully dissected and excised. Then 200-300 μL of sterile saline was added to every 0.5 g of tissue to prevent dehydration. The tissue was cut into < 1 mm^3 ^size pieces using a sharp, sterile surgical scissors. Sterile saline (37°C) was added to the homogenized adipose tissue in a ratio of 3:1 (saline: adipose tissue), followed by the addition of stock collagenase solution to a final concentration of 0.5 Units/mL. The tubes with the contents were placed and secured on a Thermaline shaker and incubated with constant agitation for 60 ± 15 min at 37°C. After 40 minutes of incubation, the content was triturated with a 25 mL pipette for 2-3 min. The cells obtained were placed back to the rocker for incubation. The contents of the flask were transferred to 50 mL tubes after digestion, followed by centrifugation at 600 g, for 5 minutes at room temperature. The fat layer and saline supernatant from the tube were poured out gently in one smooth motion or removed using vacuum suction. The cell pellet thus obtained was resuspended in 40 mL saline and then centrifuged again at 600 g for 5 minutes at room temperature. After being resuspended again in 5 mL saline, the cell suspension was filtered through a 100 μm filter into a 50 mL conical tube to which 2 mL of saline was added to rinse the remaining cells through the filter. The flow-through was pipetted to a 40 μm filter into a new 50 mL conical tube. The tubes were centrifuged for a third time at 600 g for 5 minutes at room temperature. The cells were resuspended in saline. An aliquot of cell suspension was then removed for cell culture in DMEM-low glucose medium contain 10% FBS for two weeks. Flow cytometric analysis was performed for identification of cellular characteristics after cell-labeling with appropriate antibodies 30 minutes before transplantation (Figure 1).

### ADMSCs Labeling Before Autologous Transplantation

Thirty min prior to autologous transplantatting ADMSCs, CM-Dil (Vybrant™ Dil cell-labeling solution, Molecular Probes, Inc.) (50 μg/ml) was added to the culture medium. This highly lipophilic carbocyanine dye, which has properties of low cytotoxicity and high resistance to intercellular transfer, can be added directly to normal culture media to uniformly label suspended or attached culture cells for their visibility in a brain infarct area (BIA) due to its distinctive fluorescence.

### Animal Model of Acute Ischemic Stoke

Pathogen-free, adult male Sprague-Dawley (SD) rats, weighing 300-350 g (Charles River Technology, BioLASCO Taiwan Co., Ltd., Taiwan) were utilized in this study. After adipose-derived mesenchymal stem cells (ADMSCs) were cultured for two weeks, acute stroke was induced in the animals. After exposure of the left common carotid artery (LCCA) through a transverse neck incision, a small incision was made on the LCCA through which a nylon filament (0.28 mm in diameter) was advanced into the distal left internal carotid artery for occlusion of left middle cerebral artery (LMCA) to induce brain infarction of its supplying region. Three hours after occlusion, the nylon filament was removed, followed by closure of the muscle and skin in layers.

### In Vivo Treatment Protocol

Ten healthy rats served as normal controls (group 1). The rats with acute IS were divided into group 2 (acute IS treated with 1 mL intravenous physiological saline at 0, 12 and 24 h after IS induction, n = 15) and group 3 [acute IS plus intravenous ADMSCs (2.0 × 10^6 ^in 0.5 cc culture medium for each time) given at 0, 12 and 24 h after IS induction, n = 15). Five rats in groups 2 and 3 were utilized for determining the brain infarct size. The sensorimotor functional test (Corner test) was performed by blinded investigators for each rat on days 0, 1, 3, 7, 14 and 21 after acute IS induction as previously described [21].

### Cellular Proliferation Test

To evaluate whether ADMSC treatment promotes cellular proliferation in the BIA, 5-bromodeoxyuridine (BrdU) was intravenously given in all three groups of animals on days 3, 5, 7, 9, and 12 after acute IS induction for labeling the proliferating cells.

### Specimen Collection

Rats in groups 1, 2, and 3 were euthanized on day 21 after IS induction, and brain in each rat was rapidly removed and immersed in cold saline. For immunohistofluorescence (IHF) study, the brain tissue was rinsed with PBS, embedded in OCT compound (Tissue-Tek, Sakura, Netherlands) and snap-frozen in liquid nitrogen before being stored at -80°C. For immunohistochemical (IHC) staining, brain tissue was fixed in 4% formaldehyde and embedded in paraffin.

### Measurement of Brain Infarct Area

To evaluate the impact of ADMSC treatment on brain infarction, coronal sections of the brain were obtained from five extra animals in group 2 and group 3 (n = 5 for each group) as 2 mm slices. Each cross section of brain tissue was then stained with 2% 3,5-Triphenyl-2H-Tetrazolium Chloride (TTC)(Alfa Aesar) for BIA analysis. Briefly, all brain sections were placed on a tray with a scaled vertical bar to which a digital camera was attached. The sections were photographed from directly above at a fixed height. The images obtained were then analyzed using Image Tool 3 (IT3) image analysis software (University of Texas, Health Science Center, San Antonio, UTHSCSA; Image Tool for Windows, Version 3.0, USA). Infarct area was observed as either whitish or pale yellowish regions. Infarct region was further confirmed by microscopic examination. The percentages of infarct area were then obtained by dividing the area with total cross-sectional area of the brain.

### TUNEL Assay for Apoptotic Nuclei

For each rat, 6 sections of BIA were analyzed by an in situ Cell Death Detection Kit, AP (Roche) according to the manufacturer's guidelines. Three randomly chosen high-power fields (HPFs) (×400) were observed for terminal deoxynucleotidyl transferase-mediated 2'-deoxyuridine 5'-triphosphate nick-end labeling (TUNEL)-positive cells. The mean number of apoptotic nuclei per HPF for each animal was obtained by dividing the total number of cells with 18.

### IHC Staining for Cellular Proliferation and Glial Fibrillary Acid Protein (GFAP)

Paraffin sections (5 μm thick) with BIA were obtained from each rat. To block the action of endogenous peroxidase, the sections were initially incubated with 3% hydrogen peroxide for 15 minutes, and then further processed using Beat Blocker Kit (invitrogen, #50-300) with immersion in solutions A and B for 30 minutes and 10 minutes at room temperature, respectively. Rabbit polyclonal antibody (1:500 dilution at 4°C overnight) against glial fibrillary acid protein (GFAP) (Dako) and monoclonal antibody (1:200 dilution at 4°C overnight) against 5-Bromo-2-DeoxyUridine (BrdU) (Sigma), were used as primary antibodies. The anti-rabbit HRP (Zymed) (1:3 dilution at room temperature for 10 minutes) for GFAP and anti-mouse HRP (Zymed) (1:3 dilution at room temperature for 10 minutes) were used as secondary antibodies, followed by application of SuperPicTure™ Polymer Detection Kit (Zymed) for 10 minutes at room temperature. Finally, the sections were counterstained with hematoxylin. For negative control experiments, primary antibodies were omitted.

### Western Blot Analysis for CXCR4 and Stromal Cell-Derived Factor-1 in BIA

Equal amounts (60 μg) of protein extracts from BIA were loaded and separated by SDS-PAGE using 12-13% acrylamide gradients. Following electrophoresis, the separated proteins were transferred electrophoretically to a polyvinylidene difluoride (PVDF) membrane (Amersham Biosciences). Nonspecific proteins were blocked by incubating the membrane in blocking buffer (5% nonfat dry milk in T-TBS containing 0.05% Tween 20) overnight for CXCR4 and one hour for stromal cell-derived factor (SDF)-1, respectively. The membranes were incubated with the indicated primary antibodies (CXCR4, 1:1000, Abcam, Actin 1:10000, Chemicon; SDF-1, 1:1000, Cell Signaling) for one hour at room temperature for CXCR4 and overnight at 4°C for SDF-1, respectively. Horseradish peroxidase-conjugated anti-rabbit immunoglobulin IgG (1:2000, Cell Signaling) was applied as the secondary antibody for one hour for CXCR4 and 45 minutes for SDF-1 at room temperature. The washing procedure was repeated eight times within an hour, and immunoreactive bands were visualized by enhanced chemiluminescence (ECL) (Amersham Biosciences) and exposure to Biomax L film (Kodak). For quantification, digitized ECL signals were analyzed using Labwork UVP software.

### Protocol for RNA Extraction

Lysis/binding buffer (High Pure RNA Tissue Kit, Roche, Germany) 400 μL and an appropriate amount of frozen brain tissue were added to a nuclease-free 1.5 mL microcentrifuge tube, followed by disruption and homogenization of the tissue by using a rotor-stator homogenizer (Roche).

For each isolation, 90 μL DNase incubation buffer was pipetted into a sterile 1.5 mL reaction tube, 10 mL DNase I working solution was then added, mixed and incubated for 15 minutes at 25°C. Washing buffer I 500 μL was then added to the upper reservoir of the filter tube, which was then centrifuged for 15 seconds at 8,000 *g*. Washing buffer II 300 μL was added to the upper reservoir of the filter tube, which was centrifuged for 2 minutes full-speed at approximately 13,000 *g*. Elution Buffer 100 μL was added to the upper reservoir of the filter tube; the tube assembly was then centrifuged for one minute at 8,000 *g *resulting in eluted RNA in the microcentrifuge tube.

### Real-Time Quantitative PCR Analysis

Real-time polymerase chain reaction (RT-PCR) was conducted using LightCycler TaqMan Master (Roche, Germany) in a single capillary tube according to the manufacturer's guidelines for individual component concentrations. Forward and reverse primers were each designed based on individual exons of the target gene sequence to avoid amplifying genomic DNA.

During PCR, the probe was hybridized to its complementary single-strand DNA sequence within the PCR target. As amplification occurred, the probe was degraded due to the exonuclease activity of Taq DNA polymerase, thereby separating the quencher from reporter dye during extension. During the entire amplification cycle, light emission increased exponentially. A positive result was determined by identifying the threshold cycle value at which reporter dye emission appeared above background.

### Immunohistofluorescence (IHF) analysis for CXCR4, SDF-1, Doublecortin, and von Willebrand Factor (vWF)

Serial cryosections (7 μm thick) with an average distance of 5 μm apart were collected from the BIA. The sections were fixed in acetone for 15 minutes at -20°C. For reducing the background, 200 μL of signal enhancer was utilized for blocking non-specific signals at room temperature for 30 minutes. IHF staining was performed using primary antibody (rabbit polyclonal antibody 1:200 dilution, at 4°C, overnight) (Santa Cruz) for CXCR4, followed by the addition of anti-rabbit Alexa Fluor 488 FITC (Molecular Probes) secondary antibody (1:200 dilution at room temperature for 30 minutes). Additionally, rabbit polyclonal antibody (1:500 dilution at 4°C overnight) (Santa Cruz) was used as primary antibody for SDF-1, followed by the addition of anti-rabbit Alexa Fluor 594 Rodamin (Molecular Probes) secondary antibody (1:200 dilution at room temperature for 30 minutes). Moreover, goat polyclonal antibody (1:50 dilution, at 4°C overnight) (Santa Cruz) was used as primary antibody to recognize doublecortin, followed by anti-goat Alexa Fluor 568 Rodamin (Molecular Probes) secondary antibody (1:200 dilution at room temperature for 30 minutes). Furthermore, rabbit polyclonal antibody (1:200 dilution at 4°C overnight) (Chemicon) was used as primary antibody against vWF, followed by anti-rabbit Alexa Fluor 488 FITC (Molecular Probes) secondary antibody (1:200 dilution at room temperature for 30 minutes). For negative control experiments, the primary antibodies were omitted. The sections were counterstained with 4', 6-Diamidino-2-phenylindole (DAPI) (dilution 1/500) (Sigma) to identify cellular nuclei that represented the cell number.

### Oxidative Stress of BIA

The Oxyblot Oxidized Protein Detection Kit was purchased from Chemicon (S7150). The 2,4-dinitrophenylhydrazine (DNPH) derivatization was carried out on 6 μg of protein for 15 minutes according to manufacturer's instructions. One-dimensional electrophoresis was carried out on 12% SDS/polyacrylamide gel after DNPH derivatization. Proteins were transferred to nitrocellulose membranes which were then incubated in the primary antibody solution (anti-DNP 1: 150) for 2 hours, followed by incubation with second antibody solution (1:300) for one hour at room temperature. The washing procedure was repeated eight times within 40 minutes. Immunoreactive bands were visualized by enhanced chemiluminescence (ECL; Amersham Biosciences) which was then exposed to Biomax L film (Kodak). For quantification, ECL signals were digitized using Labwork software (UVP). On each gel, a standard control was loaded.

### Small Vessel Density in BIA

IHC staining of small blood vessels (i.e. diameters ≤ 15 mm) was performed with anti-α-SMA (1:400) as primary antibody at room temperature for one hour, followed by washing with PBS thrice. The anti-mouse HRP-conjugated secondary antibody was then added and incubated for 10 minutes, followed by washing with PBS thrice. Then 3,3' diaminobenzidine (DAB) (0.7 gm/tablet) (Sigma) was added and incubated for one minute, followed by washing with PBS thrice. Finally, following hematoxylin treatment for one minute as a counter stain for nuclei, the sections were washed twice. Three coronal sections of the brain were analyzed in each rat. For quantification, three randomly selected HPFs (200×) were analyzed in each section. The mean number of small vessel per HPF for each animal was then determined by summation of all numbers divided by 9.

### Statistical Analysis

Data were expressed as mean values (mean ± SD). The significance of differences between two groups was evaluated with *t*-test. The significance of differences among three groups was evaluated using analysis of variance followed by Bonferroni multiple-comparison post hoc test. Statistical analyses were performed using SAS statistical software for Windows version 8.2 (SAS institute, Cary, NC). A probability value < 0.05 was considered statistically significant.

## Results

### ADMSC Therapy Limited Brain Infarct Size and Enhanced Recovery of Neurological Function (Figure 2)

**Figure 2:**
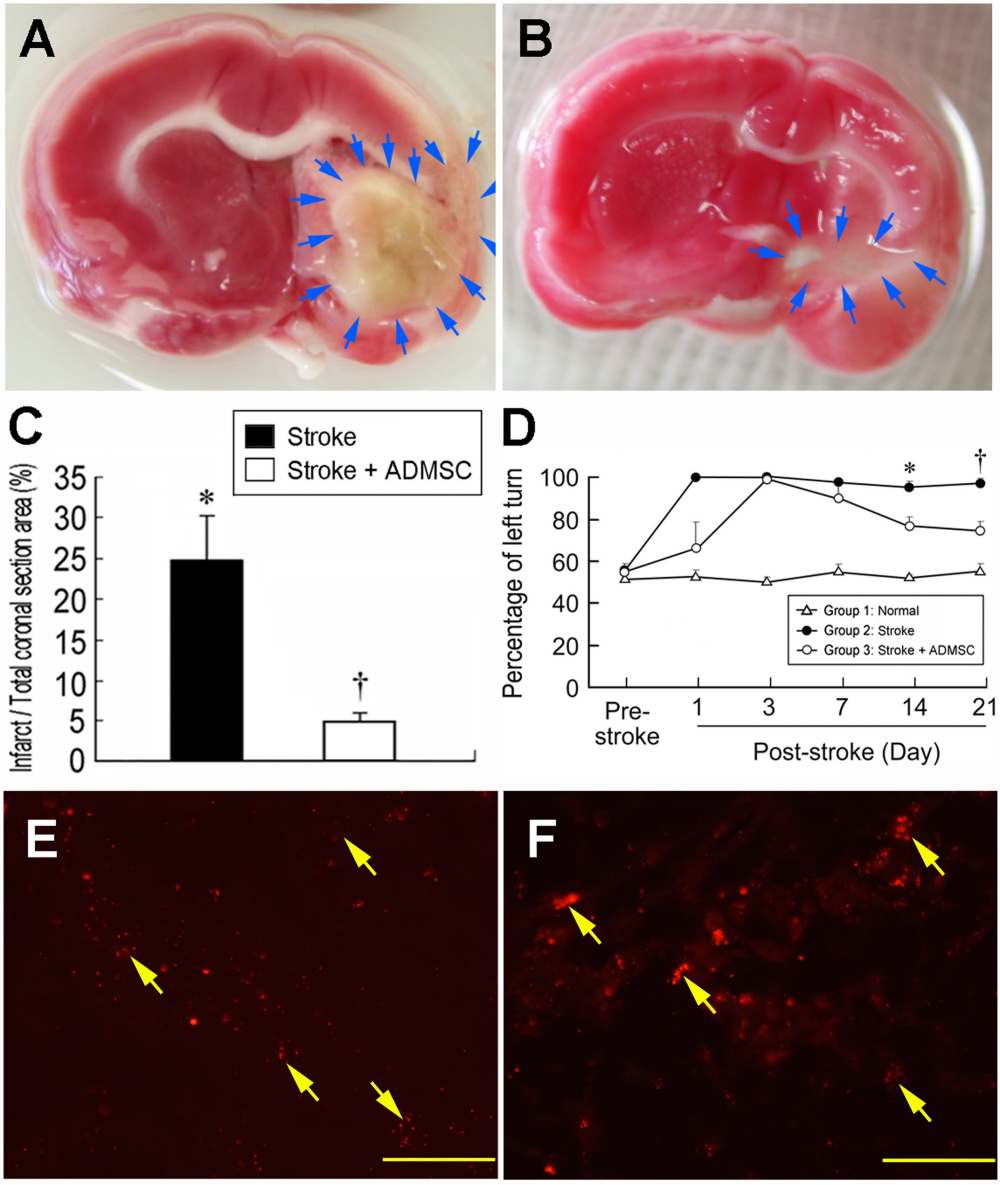
**Comparison of infarct area and sensorimotor function in rats with and without ADMSC treatment. **(**A & B**) Identification of gross infarct area (blue arrows) with and without ADMSC treatment, respectively. (**C**) Significantly lower ratio of infarct area to total coronal section area in stroke + ADMSC group (group 3) compared with stroke group (group 2) (n = 5 per group). † vs. *, p < 0.001. (**D**) The results of Corner test on days 0, 3, 7, 14, and 21 after acute IS, showing a steady state of neurological functional impairment on day 3 following acute IS in both group 2 and group 3. Significant improvement in neurological function noted only in group 3 compared with group 2 on day 14 and substantially improved on day 21 after acute IS. Significance of difference at respective time point: * p < 0.005, group 2 vs. group 3; † p < 0.002, group 2 vs. group 3. (**E**) and (**F**) Identification of CM-DiI-stained ADMSCs (yellow arrows) in brain infarct area of two rats from group 3. Scale bars in right lower corner represent 50 μm.

TTC staining on day 21 after acute IS showed a markedly larger BIA in IS animals without treatment (group 2) compared with those having received ADMSC therapy (group 3) (Figure 2A-C). Additionally, corner test demonstrated a steady state of neurological functional impairment on day 3 following acute IS in both group 2 and group 3 (Figure 2D). On the other hand, progressive improvement in neurological function after day 3 became significant on day 14 in group 3 but not in group 2. Moreover, substantial improvement in group 3 was noted on day 21 while persistent impairment of neurological function was observed in group 2 after acute IS.

By day 21 following ADMSCs implantation, immunofluorescence stain (Figure 2E-F) identified that numerous CM-Dil-stained ADMSCs were found to be present in infarct area. This finding indicates that ADMSCs was able to migrate (i.e. homing) to brain infarcted area after venous injection.

### Autologous Transplantation of ADMSCs Attenuated Anti-Inflammatory Response, Apoptosis, and Oxidative Stress (Figures 3, 4 and 5)

**Figure 3:**
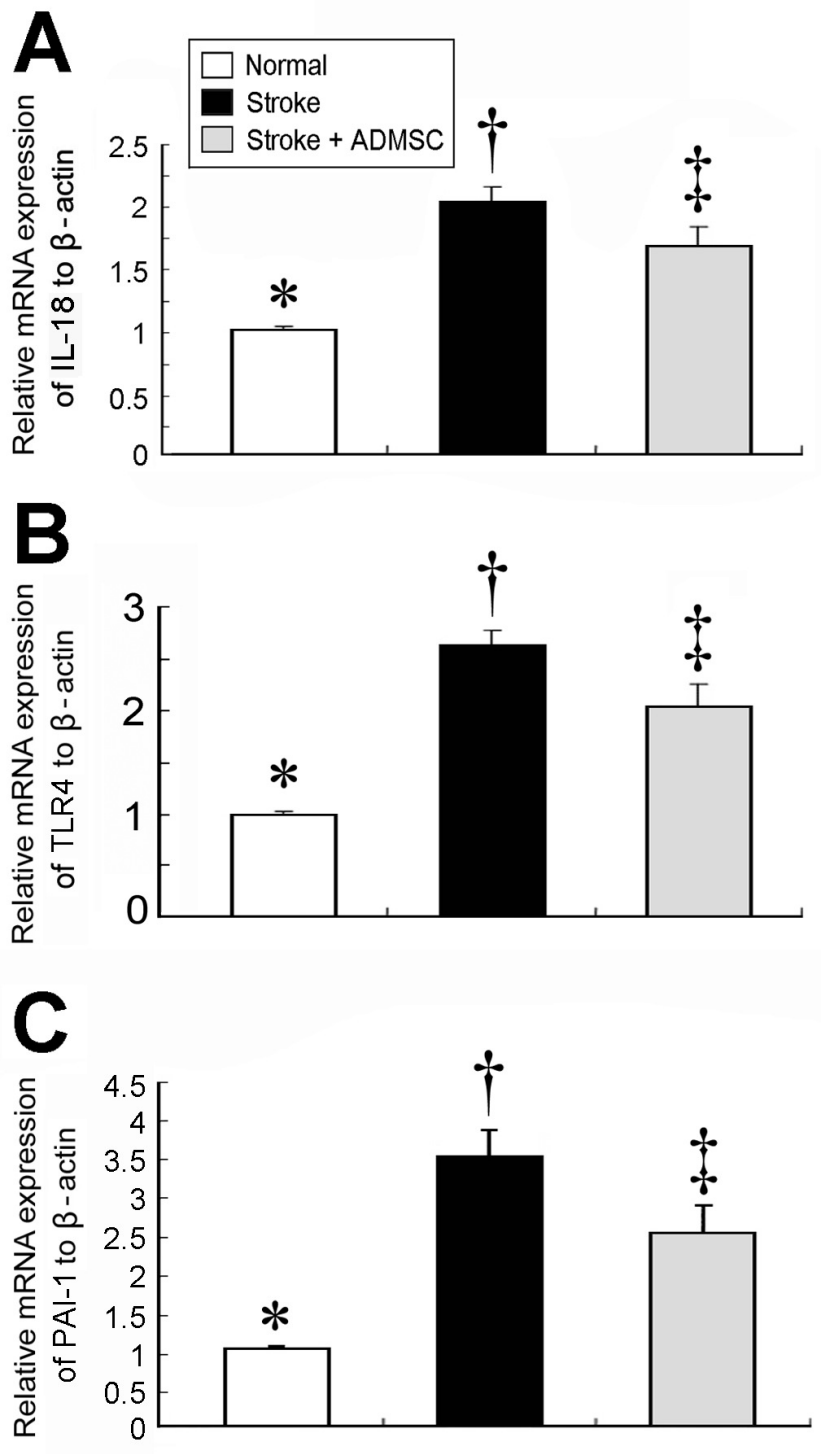
**mRNA expressions of inflammatory mediators in brain infarct area.** Significantly higher mRNA expressions of **(A) **interleukin (IL)-18, **(B) **toll-like receptor (TLR)-4, and **(C) **plasminogen activator inhibitor (PAI)-1 in group 2 than in group 3 and normal controls (group 1), and notably higher in group 3 than in group 1. (n = 10 per group) * vs. †, p < 0.001; * vs. ‡, p < 0.01; † vs. ‡, p < 0.04.

**Figure 4:**
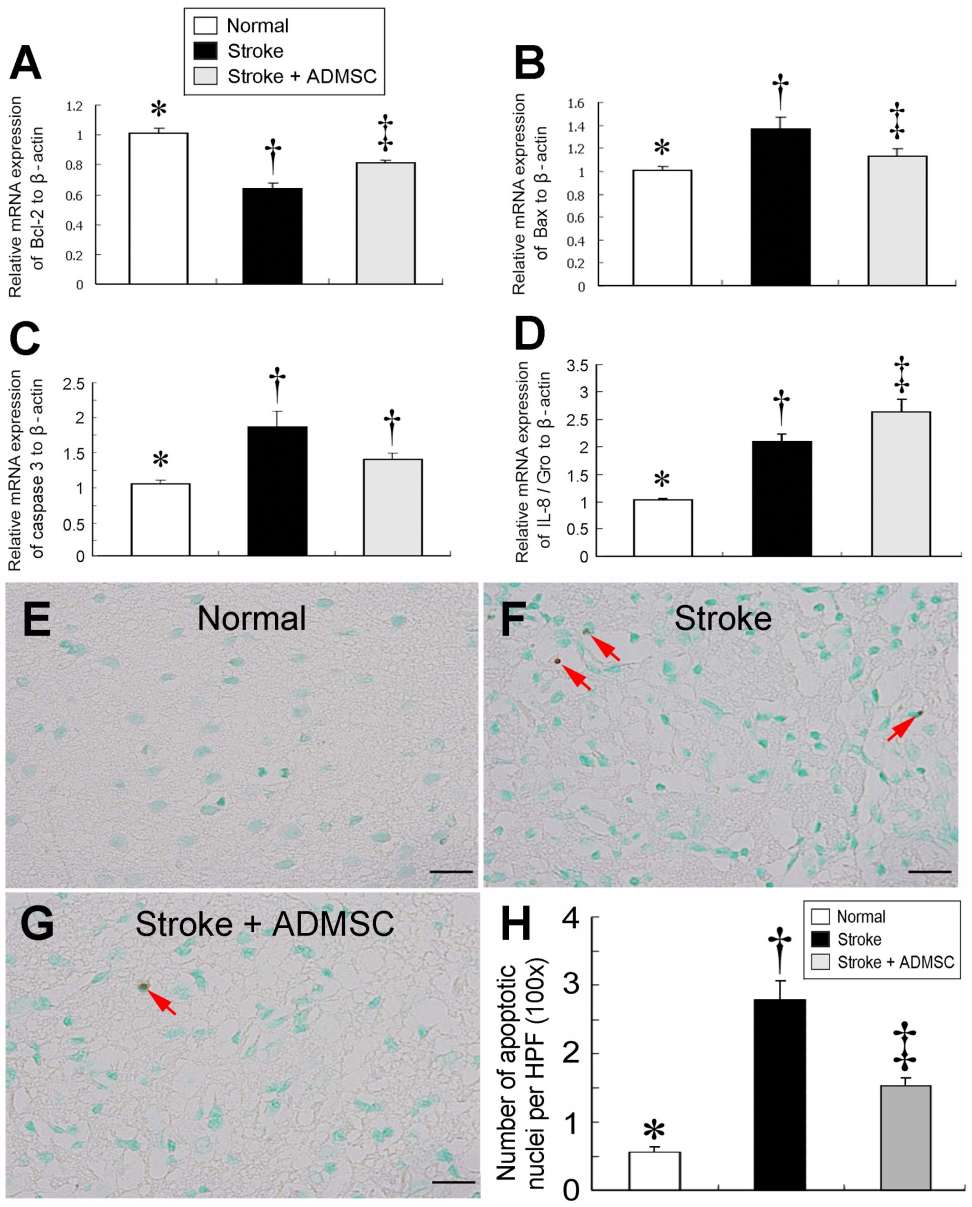
**mRNA expressions of apoptosis-related genes and number of apoptotic cells in brain infarct area. (A) **Bcl-2 mRNA expression was significantly higher in groups 1 and 3 than in group 2 and notably higher in group 1 than in group 3. **(B) **Bax mRNA expression was notably higher in group 2 than in groups 1 and 3 and significantly higher in group 3 than in group 1. **(C) **Caspase 3 mRNA significantly higher in groups 2 and 3 than in group 1, but it did not differ between group 2 and group 3. **(D) **IL-8/Gro mRNA expression was remarkably higher in groups 2 and 3 than in group 1 and notably higher in group 3 than in group 2. * vs. †, p < 0.05; * vs. ‡, p < 0.05; † vs. ‡, p < 0.05. The number of apoptotic nuclei **(H)) **(400×) significantly higher in group 2 **(F) **than in groups 1 **(G) **and 3 **(E)**, and notably higher in group 3 than in group 1. (n = 10 per group) * vs. † vs. ‡, p < 0.001. Scale bar in right lower corner represent 20 μm.

**Figure 5:**
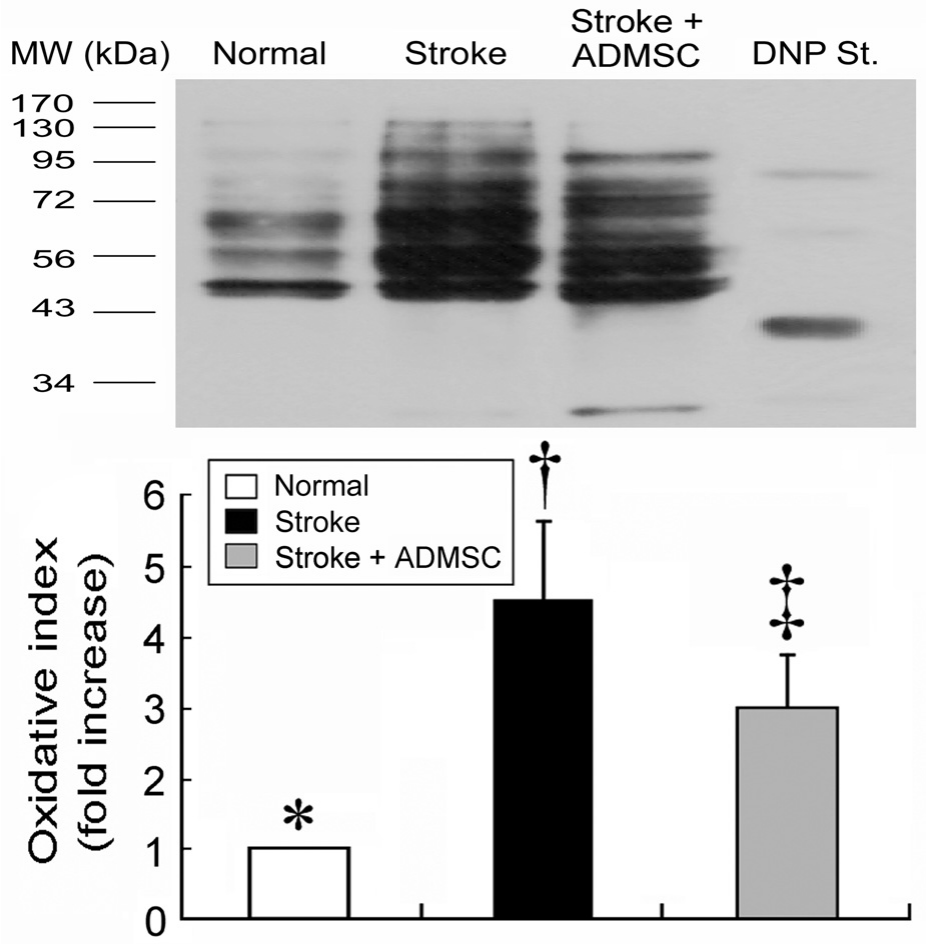
**Oxidative index in brain infarct area.** Western blotting showing notably increased oxidative index, protein carbonyls, in BIA of group 2 compared with groups 1 and 3 on day 21 following acute IS (upper panel), with quantification results of each group (n = 10) shown (lower panel). * vs. † vs. ‡, p < 0.009.

On day 21 following acute IS induction, mRNA expressions (Figure 3) of interleukin-18 (IL-18), toll-like receptor (TLR)-4, and plasminogen activator inhibitor (PAI)-1 in BIA, indexes of inflammation, were significantly elevated in group 2 compared with groups 1 and 3, and significantly lower in group 1 than in group 3. These findings indicate that ADMSC therapy attenuated inflammatory reaction.

On day 21 following acute IS induction, Bcl-2 mRNA expression, an anti-apoptotic index, was significantly reduced in group 2 compared with groups 1 and 3, and notably higher in group 1 than in group 3 (Figure 4A), whereas mRNA expressions of Bax, an index of apoptosis, was significantly elevated in group 2 compared with groups 1 and 3, and notably lower in group 1 than in group 3 (Figure 4B). Additionally, caspase 3 mRNA expression, another indicator of apoptosis, was also remarkably lower in group 1 than in groups 2 and, but it did not differ between group 2 and group 3 (Figure 4C). Furthermore, mRNA expression of IL-8/Gro, which has been shown to regulate stem cells homing in response to ischemic stress,^21 ^was substantially higher in group 3 than group 2 (Figure 4D). Moreover, TUNEL assay showed a significantly reduced number of apoptotic nuclei in group 3 than in group 2 (Figure 4E-H).

On day 21, Western blotting (Figure 5) demonstrated that oxidative stress index in mitochondria was markedly elevated in group 2 compared with that in groups 1 and 3, and notably lower in group 1 than in group 3. These findings indicate that ADMSC transplantation exerted both anti-apoptotic and anti-inflammatory actions in the brain after IS.

### Autologous Transplantation of ADMSCs Enhanced In Vivo Angiogenesis and Neurogenesis (Figures 6, 7, 8, and 9)

**Figure 6:**
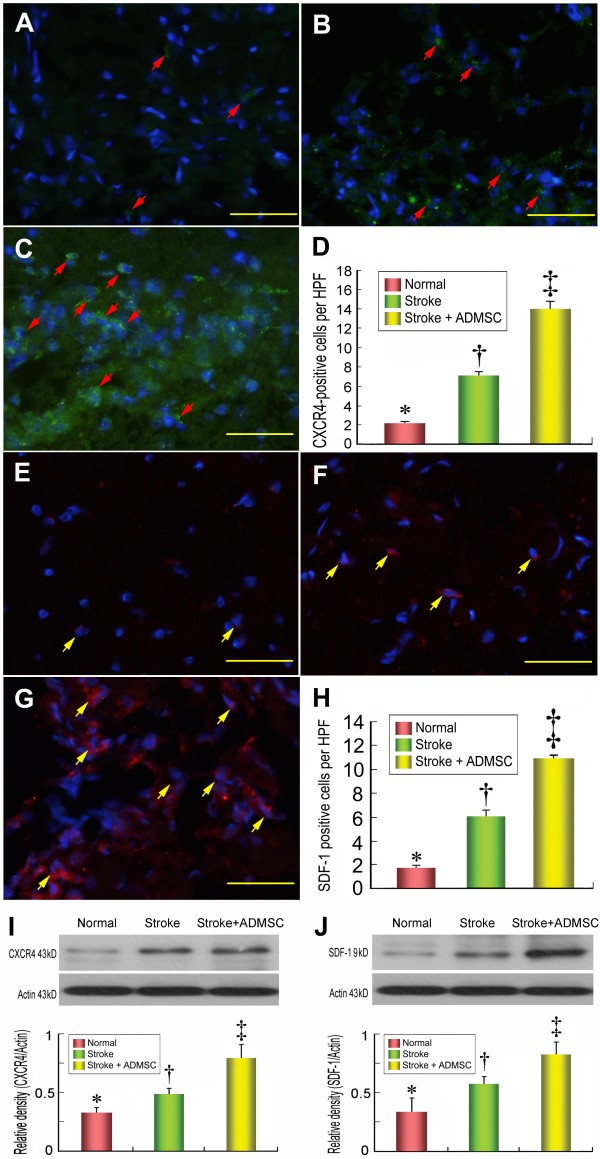
**Cellular expressions of CXCR4 and stromal derived factor (SDF)-1 in brain infarction area**. Immunohistofluorescence (IHF) staining (400×) showing substantially lower number of CXCR4-positive cells (red arrows) in group 1 **(A) **than in groups 2 **(B) **and 3 **(C)**, and remarkably lower in group 2 than in group 3; **(D) **indicated quantification results of each group (n = 10). ‡ vs. † vs. *, p < 0.001. IHF staining (400×) also demonstrating significantly lower number of SDF-1 positive cells (yellow arrows) in group 1 **(E) **than in groups 2 **(F) **and 3 **(G)**, and notably lower in group 2 than in group 3. **(H) **indicated quantification results of each group (n = 10). ‡ vs. † vs. *, p < 0.001. Western blotting showing markedly lower CXCR4 **(I) **and SDF-1 **(J) **protein expressions in group 1 than in groups 2 and 3, and notably lower in group 2 than in group 3. n = 10 per group. Scale bars in right lower corner represent 50 μm. * vs. † vs. ‡, p < 0.01.

**Figure 7:**
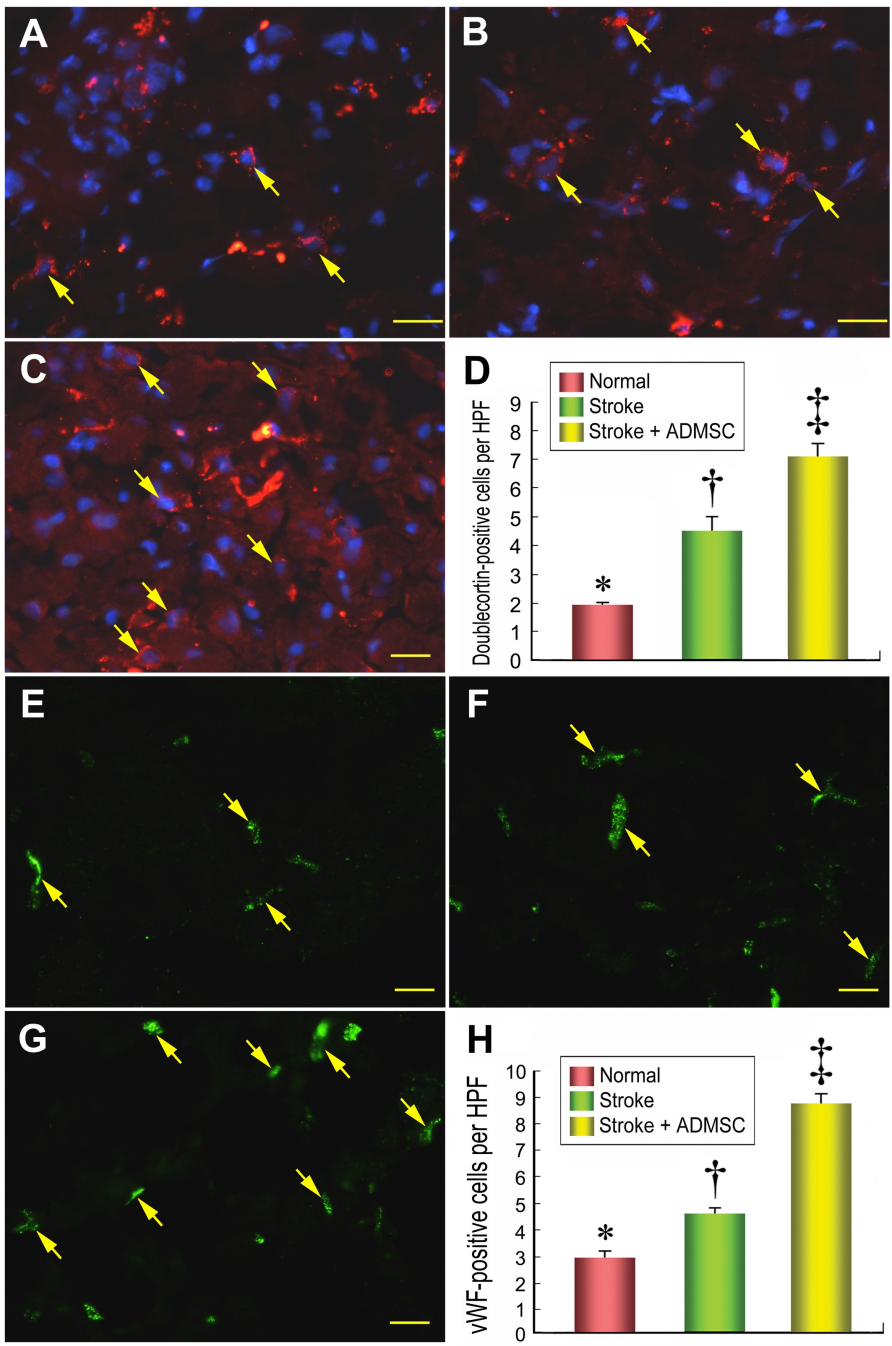
**The distribution of neuroblasts and endothelial cells in brain infarction area**. Results of IHF staining **(D) **(400×) showing significantly lower number of doublecortin-positive cells (yellow arrows) in group 1 **(A) **than in groups 2 **(B) **and 3 **(C)**, and significantly lower in group 2 than in group 3. IHF staining **(H) **(400×) demonstrating significantly lower number of von Willibrand factor (vWF)-positive cells (yellow arrows) in group 1 **(E) **than in groups 2 **(F) **and 3 **(G)**, and notably lower in group 2 than in group 3 **(H)**. n = 10 in each study group. Scale bars in right lower corner represent 20 μm. * vs. † vs. ‡, p < 0.001.

**Figure 8:**
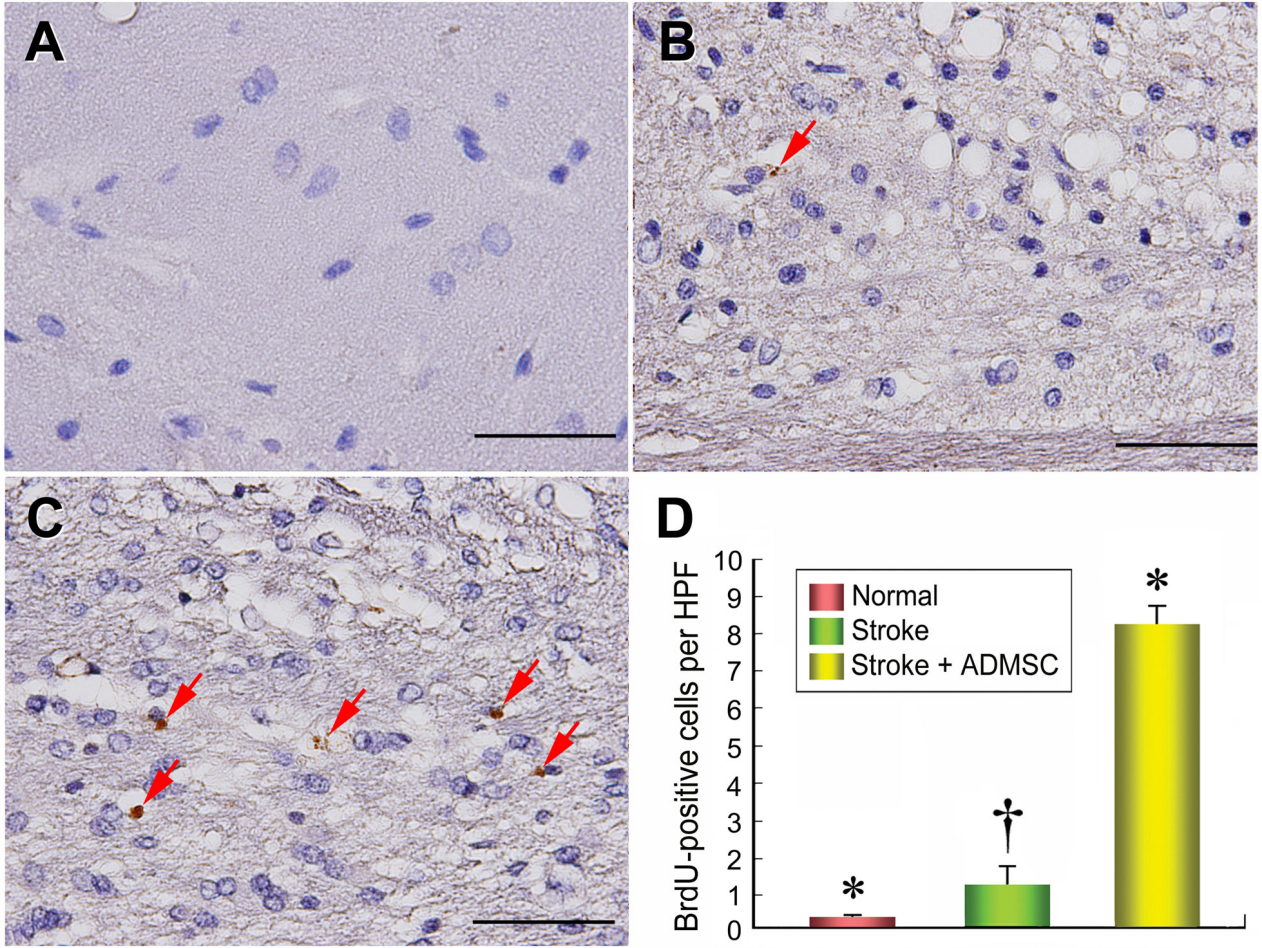
**The distribution of proliferative cells in brain infarction area**. Immunohistochemical (IHC) staining **(D) **(400×) showing markedly lower number of 5-bromodeoxyuridine (BrdU)-positive cells (red arrows) in groups 1 **(A) **and 2 **(B) **than in group 3 **(C)**. No difference of BrdU-positive cells between groups 1 and 2. n = 10 in each group. Scale bars in right lower corner represent 50 μm. * vs. †, p = 0.096; * vs. ‡, p < 0.0001; † vs. ‡, p < 0.0001.

**Figure 9:**
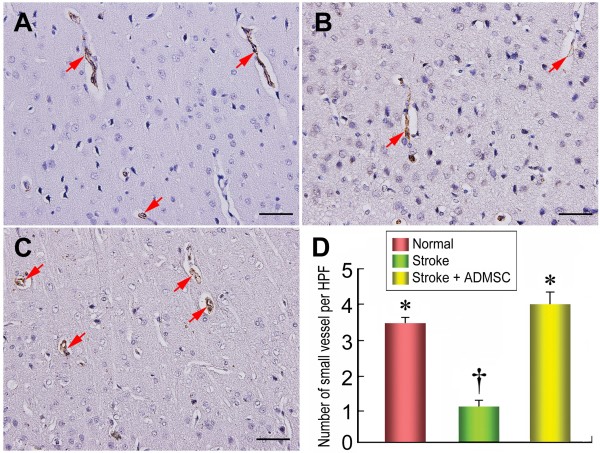
**The number of arterioles in brain infarction area**. Small vessels (diameters ≤ 15 mm) (red arrows) quantification for each group (n = 10) on 21 day following acute IS **(D)**. Identification of blood vessel distribution in BIA using α-SMA immunohistochemical staining, showing notably higher small vessel number in groups 1 **(A) **and 3 **(C) **than in group 2 **(B) **(200×), and similar between groups 3 and 1. Scale bars in right lower corner represent 50 μm. † vs. *, p < 0.0001.

IHC staining demonstrated that the number of cells positive for CXCR4 (Figure 6A-D), a surface cell marker of endothelial progenitor cells (EPCs), and SDF-1, a chemokine for attraction of EPCs having CXCR4 receptor (Figure 6E-H), was significantly higher in group 3 than in group 2, suggesting an enhancement of circulating EPC homing to ischemic area of the brain following ADMSC treatment. Consistently, Western blot analysis revealed significantly higher protein expressions of CXCR4 (Figure 6I) and SDF-1 (Figure 6J) in group 3 than in group 2.

The expression of doublecortin, an indication of migrating neuroblasts, was remarkably upregulated in group 3 compared with group 2 (Figure 7A-D). Additionally, IHC staining showed that the expression of vWF, a marker of endothelial cells of cerebral blood vessels, was significantly increased in group 3 than in group 2 (Figure 7E-H). Moreover, IHC staining also revealed a notably increased number of BrdU-positive cells (Figure 8A-D) in group 3 than in group 2, implying an increased cellular differentiation and proliferation after ADMSC treatment. Furthermore, the number of arterioles (≤ 15 μm in diameter) in BIA was substantially lower in group 2 than in groups 1 and 3 on IHC staining (Figure 9A-D). All of these findings indicate an ADMSC-induced enhancement in neurogenesis and vasculogenesis after acute IS.

### Autologous ADMSC Transplantation Reduced Glial Fibrillary Acid Protein (GFAP) Expression in Infarcted Brain (Figure 10)

**Figure 10:**
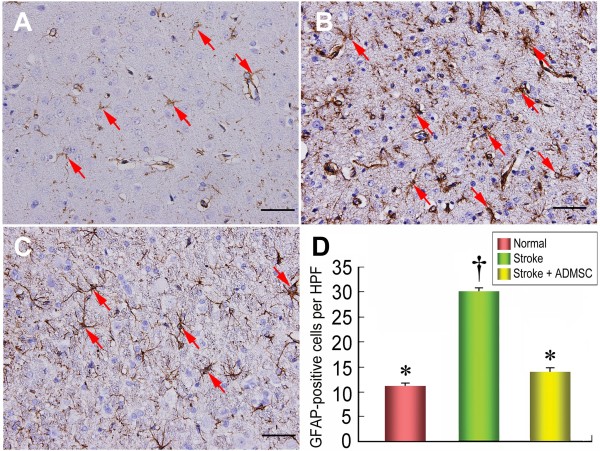
**The number of GFAP-positive cells in brain infarct area. **IHC staining **(D) **(200×) showing significantly higher number of glial fibrillary acid protein (GFAP)-positive cells (red arrows) in group 2 **(B) **than in groups 1 **(A) **and 3 **(C)**, and no difference between groups 1 and 3. n = 10 per group. Scale bars in right lower corner represent 50 μm. * vs. †, p < 0.0001.

IHC staining showed that GFAP expression, the principal intermediate filament of mature astrocytes, was notably lower in group 3 than in group 2 (Figure 10A to 10D), suggesting reduced IS-induced gliosis after ADMSC treatment.

### Double Stains of CM-Dil and DAPI, and CM-Dil and vWF (Figure 11)

**Figure 11:**
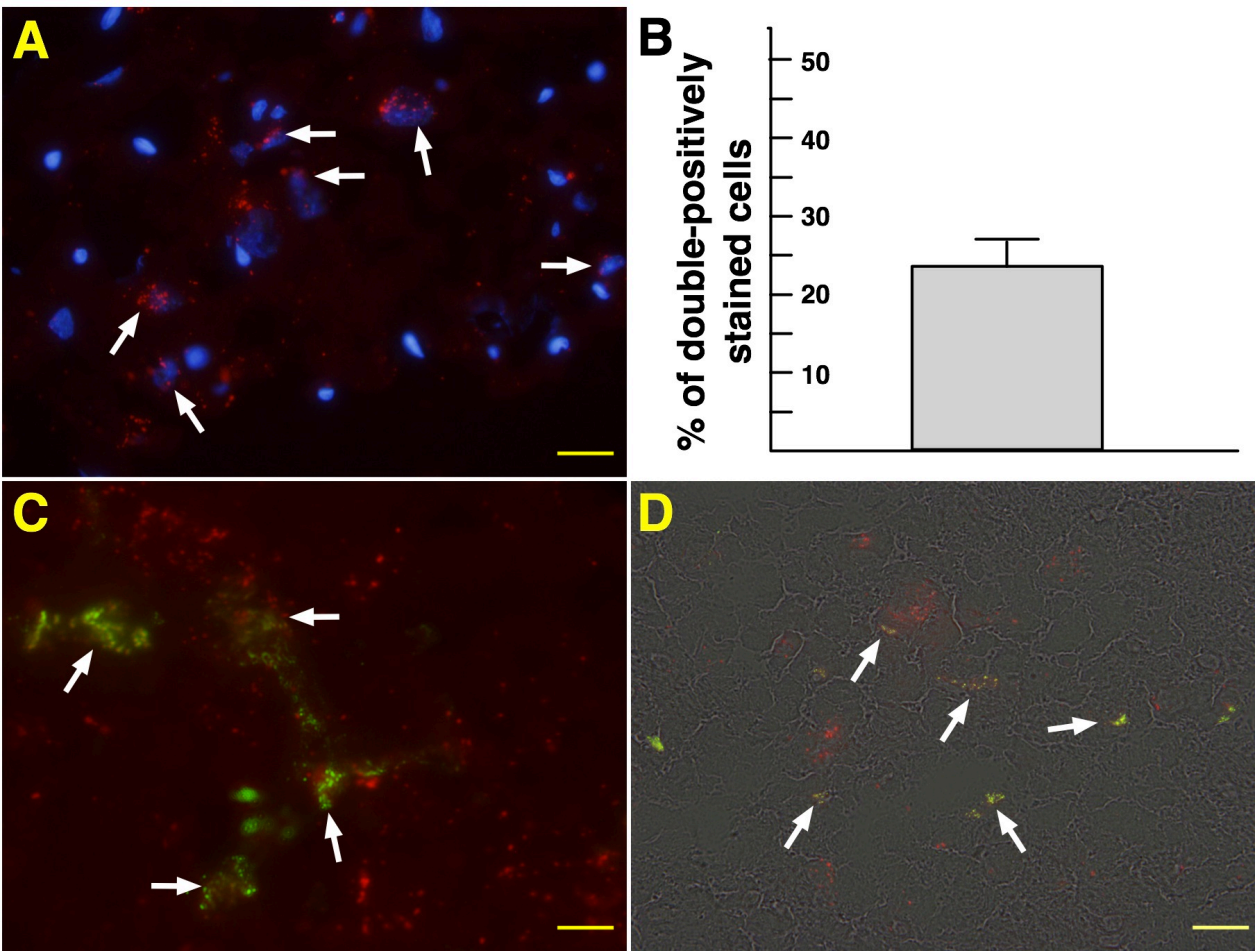
**Identification of ADMSCs in brain infarct area and differentiation into endothelial-cell phenotype**. The IHF imaging **(A) **(400×) results showing double stains of CM-Dil and DAPI-positive cells (white arrows) in the brain infarct area. The number of these double stained cells expressed as percentage **(B)**. The merge results of IHF (400×) showing the double stain of CM-Dil and vWF-positive cells **(C) **(white arrows) and bright field **(D) **image (800×) further confirmed the presence of these cells (white arrows). Scale bars in right lower corner represent 20 μm in (A) and (D) and 10 μm in (D).

To determine whether transplanted ADMSCs were really homing to the BIA, double stain of CM-Dil and DAPI was done. As expected, the CM-Dil and DAPI-positively stained cells were found to engraft into the BIA (Figure 11A). Additionally, to evaluate whether the implanted ADMSCs were able differentiation into endothelial cell phenotype, double stain of CM-Dil and vWF was performed. The results showed that some of the CM-Dil and vWF-positively cells were found to integrate into small vessels (Figure 11C-D). This finding implicates that some of ADMSCs might differentiate into endothelial cells.

## Discussion

This study, which investigated whether ADMSC therapy limited brain infarct size and promoted neurological recovery in a rat model of acute IS, produces several important findings. First, ADMSC therapy enhanced angiogenesis/vasculogenesis and neurogenesis. Second, ADMSC therapy attenuated inflammatory reaction and apoptosis in BIA. Third, ADMSC therapy significantly limited brain infarct size and improved neurological outcome.

### Limitation and Prospect of Stem Cell Therapy for Patients after Acute Ischemic Stroke

The preliminary results of stem cell therapy appear to be promising for stroke patients in restoring sensorimotor functions [3,4,14,18,22,23]. The validity of its clinical applicability, however, depends on tangible evidence on its safety and effectiveness as well as a thorough understanding of the underlying mechanism of actions. The use of an animal model of acute IS, therefore, is imperative to investigate the short and long-term effects of such a novel treatment strategy [23]. Currently, several candidates of stem cells, including embryonic stem cell, neuron stem cells, bone marrow-derived mesenchymal stem cell, and peripheral blood-derived stem cells have been frequently investigated for their feasibility and safety in the treatment of stroke in both clinical observational studies and animal models [3,4,14,18,22-24]. However, the application of these stem cells for stroke patients is commonly hampered by a lot of limitations, including ethical problems with using embryonic stem cells, difficulties in differentiation, lineage restriction and identification as well as limitation of number and functional integrity in neuron stem cells, peripheral blood-revived stem cell, and bone marrow-derived stem cells [3,22-24]. One important finding in the present study was that the Dil dye-label ADMSCs were identified on day 21 after acute IS induction. Additionally, transplantation of ADMSCs facilitated recovery of forelimb function in the Corner test. These findings, in addition to supporting our original hypothesis, may provide one of ideal cell source, i.e. ADMSCs, for transplantation. The administration of ADMSCs through the systemic venous route has also been validated in this study. Our results, therefore, offer a potential clinical avenue for the future use of ADMSCs in IS patients.

### ADMSC Therapy Enhances Angiogenesis and Neurogenesis

SDF-1α is an endothelial progenitor cell chemokine participating in the mobilization, incorporation, homing, survival, proliferation, and differentiation of stem cells [18,25]. Recently, SDF-1α and its receptor CXCR4 are proven crucial in bone marrow retention of hematopoietic stem cells, angiogenesis, and recruitment of EPCs into ischemic tissue [18,25-29]. Another important finding in the current study was that both Western blot and IHC staining demonstrated that both CXCR4 and SFD-1 expressions were substantially increased in animals with acute IS as compared with the normal controls. These findings, therefore, are comparable to those of previous studies [25-29]. Importantly, CXCR4 and SDF-1 in BIA were found to be markedly increased after ADMSC treatment. A recent study has recently demonstrated that administration of SDF-1α to an animal model of critical limb ischemia enhances the concentrations of EPCs within the ischemic tissue and augments tissue reperfusion [28]. Taking this finding [28] into consideration, our results suggest that the enhancement of the number of CXCR4-positive cells in BIA by administration of ADMSCs may be partially through reinforcing SDF-1α chemokine expression in the BIA.

Beside the findings of upregulated expressions of CXCR4 and SDF-1α in BIA, ADMSC therapy also markedly increased the cellular expression of vWF that is a marker of endothelial cells. Importantly, ADMSC therapy also increased the number of small vessels in BIA. Taken together, the improved neurological function and reduced BIA in the present study could be explained, at least in part, by the impact of angiogenesis.

As expected, the current study revealed that administration of ADMSCs significantly increased the number of doublecortin-positive cells in BIA. Additionally, BrdU uptake in BIA, an index of cellular differentiation and proliferation, was substantially promoted following ADMSC treatment. Accordingly, the results of the present study suggest that ADMSC therapy enhances both neurogenesis and vasculogenesis. These findings could be another explanation for the reduction in BIA and improvement in neurological function.

### ADMSC Therapy Attenuates Inflammatory Response, Oxidative Stress, and Apoptosis

In the present study, the mRNA expressions of IL-18, TLR-4, and PAI-1 were markedly upregulated in rats after acute IS. In addition, the mRNA expressions of Bax and caspase 3 were remarkably increased, whereas mRNA expression of Bcl-2 was notably reduced in rats after acute IS. Furthermore, TUNEL assay and IHC staining demonstrated markedly increased number of apoptotic nuclei and GFAP-positive cells, respectively, after acute IS. Moreover, Western blot showed remarkably upregulated oxidative stress in rats after acute IS. Surprisingly, these biomarkers were significantly reversed by ADMSC therapy. Recently, Thum et al. proposed that stem cell therapy modulates immune reactivity by down-regulating innate and adaptive immunity [30]. Accordingly, our findings not only reinforce this hypothesis [30], but also account for the improvement in neurological outcome after ADMSC treatment in rats following acute IS.

### ADMSC Therapy Improves Neurological Function-Mechanisms of Uncertainty

Although the role of mesenchymal stem cell therapy in improving ischemia-related organ dysfunction have been well established [12-14,31-33], the exact mechanism remains unclear [12,32]. The proposed mechanisms, including angiogenesis [12,34] cytokine effects [12,15,34], effect of paracrine mediators [12,15,32,33], neurogenesis [14,16-18], or a stem-cell homing effect [16,35], underlying improved ischemia-related organ dysfunction following mesenchymal stem cell therapy have been extensively debated.

In the current study, although only a relatively lower percentage of implanted cells were positive for neuron surface makers, including nestin and microtubule-associated protein 2 (MAP-2), on flow cytometric analysis following 14 days of culturing, both pathological finding (TTC staining) and corner test demonstrated that intra-venous administration of ADMSCs into ischemic stroke animals significantly reduced brain IA and remarkably improved the recovery of neurological function in this study compared to animals with ischemic stroke treated by saline injection alone. Accordingly, rather than supporting a crucial role of direct cellular participation in limiting brain infarct size and improving neurological function, our findings suggest the existence of other unproved confounders.

**Figure 12:**
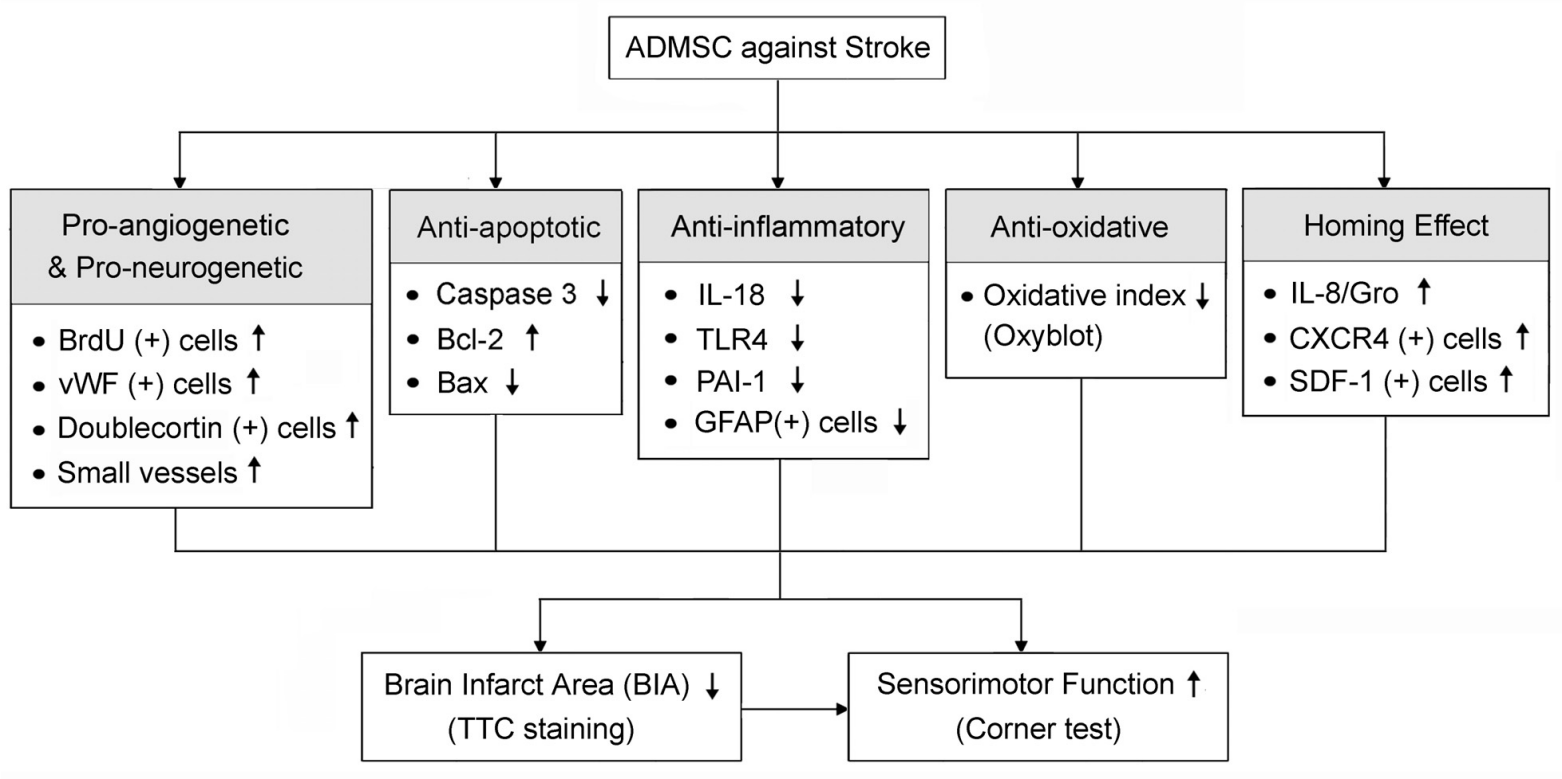
**Proposed mechanisms underlying therapeutic effects of SDMCs on reducing brain infarct area and improving neurological function**.

The present study has limitations. First, although the mechanisms underlying the therapeutic potential of ADMSC in attenuating BIA and enhancing sensorimotor functional recovery have been carefully elucidated, the precise mechanistic basis of ADMSC treatment for acute IS may be more complex. The proposed mechanisms of potential impacts of ADMSC implantation on improving sensorimotor dysfunction in the rat have been summarized in Figure 12. Second, although the short-term outcome was impressive, this current study does not provide the information for how long the therapeutic effect will be maintained.

In conclusion, ADMSC therapy limited brain infarct size and improved neurological function in rats after acute IS through enhancement of angiogenesis/vasculogenesis and neurogenesis as well as its anti-inflammatory and anti-apoptotic effects.

## Competing interests

The authors declare that they have no competing interests.

## Authors' contributions

All authors have read and approved the final manuscript. CMY and SL designed the experiment, drafted and performed animal experiments. YCL, CHY, and YHK were responsible for the laboratory assay and troubleshooting. CKS and HKY participated in refinement of experiment protocol and coordination and helped in drafting the manuscript.
